# Effect of patient age and breast parenchymal density on Breast Imaging-Reporting and Data System (BIRADS-4) Subcategorization

**DOI:** 10.12669/pjms.40.10.9552

**Published:** 2024-11

**Authors:** Hadeel A Ghunaim

**Affiliations:** Hadeel A Ghunaim, MBBS. Assistant Professor and Consultant Radiologist, Department of Radiology, College of Medicine, Taibah University, Madinah, Saudi Arabia.

**Keywords:** BI-RADS category 4, Age, Breast cancer, Breast composition, Positive predictive value

## Abstract

**Background & Objectives:**

BI-RADS (Breast Imaging-Reporting and Data System) is a standard radiological risk assessment for breast lesions, including six categories, of which category four is the widest range of likelihood cancer risk (2% to 95%). This study aimed to evaluate the effect of patient age and ACR breast density on the positive predictive value (PPV) of BI-RADS 4 subcategorization (4A, 4B, and 4C).

**Methods:**

A retrospective study was conducted at King Fahed Hospital (KFH) between September 1, 2021, and June 30, 2022. PPV was calculated based on a histopathological report for all lesions. The correlation was made with patients’ age groups ranging from < 45 years, 45-55 years, and > 55 years and four types of breast density on mammography. We used IBM-SPSS for data synthesis. The Chi-square test was used to assess any correlation between variables. A p-value < 0.05 was considered statistically significant.

**Results:**

The mean age was 44.62 ±14.32. Of the 248 cases, 81% were benign, 17% were malignant, and 2% were high-risk lesions. The age-related PPV of each BI-RADS category showed no significant differences among all age groups. However, the breast composition-related PPV cases with BI-RADS categories differed significantly within subcategory 4C (p-value<0.001).

**Conclusions:**

There were no positive relationships between increasing age and PPV in BI-RADS 4 subcategories 4A, B, and C. Breast composition-related PPV showed significant differences with BI-RADS 4C subcategories.

## INTRODUCTION

Breast cancer in women is a major cause of mortality and a global health issue. According to WHO figures, 2.3 million women were diagnosed with cancer in 2020, and 685,000 people died worldwide.^1^ The probability of developing breast cancer in a woman’s lifetime is one in four cancer cases.^1^ In our society, breast cancer cases significantly increased from 783 to 2240 cases from 2004 to 2016, respectively.^2^ Breast Imaging Report and Data System (BI-RADS) is a standardized risk estimation, imaging terminology, and report organization of breast pathology in ultrasound (US), mammography (MG), and magnetic resonance imaging (MRI) the last fifth updated edition by the American College of Radiology (ACR) in 2013.^3^ BI-RADS are categorized from 0-6, each with a corresponding risk for malignancy, management strategy, and advice. It has a significant impact on the diagnosis, with BI-RADS four providing a wide range of likelihood malignancy, > 2% but < 95% likelihood of malignancy and most pathology encounter to biopsy, therefore subcategorized to (A, B, C). Category 4A: Low suspicion, Category 4B: Moderate suspicion and Category 4C: High suspicion for malignancy, with a likelihood of malignancy > 2% to ≤ 10%, > 10% to ≤ 50% and > 50% to < 95% respectively.^3^

Breast tissue is composed of fibrous, glandular, and fatty tissue. The percentage of fibro glandular tissue in mammographs refers to breast density; BIRADS categorizes mammographic breast density into four types: A, when breasts are almost entirely fatty; B, when there are scattered areas of fibro glandular density; C, the breasts are heterogeneously dense, which may obscure small masses, and D, the breasts are extremely dense, lowering the mammography’s sensitivity.^4^,^5^

The radiologist should assign one type of density in their report to easily communicate with the referring physician and know how hard it is to read a mammogram, the sensitivity of the findings, and the risk of breast cancer. Breast density significantly increases the risk of breast cancer, reaching from 1.7 to 4.6 times.^6^ The woman’s age is an unchangeable major risk factor; Breast cancer is frequently diagnosed at age 55-64 years in American women.^7^ In Saudi Arabia (SA), the median age is 50 years, and approximately 60% of patients were diagnosed > 40 years in 2016.^2^ Other factors that increase the risk of developing breast cancer, being female, having specific gene mutations, having a personal or first-degree relative history of breast cancer, race and ethnicity, having dense breast tissue or specific benign pathologies, as well as hormonally related risk factors, chest treated with radiation therapy, and lifestyle-related risk factors.^8^

The present study is the first in SA that studies the effect of patient age and breast density on BIRADS 4 categorization since breast density is affected by race.^9^ The median age of breast cancer in Saudi women is younger than in the developed country, and there are no objectives or risk factor criteria for BIRADS 4 subcategorization. This study aims to evaluate the effect of patient age and ACR breast density on positive predictive value (PPV) of BI-RADS 4 subcategorization (4A, 4B and 4C).

## METHODS

This retrospective study was conducted in King Fahed Hospital (KFH). Data collected from the Hospital Information System between September 1, 2021 and June 30, 2022. 274 lesions were classified as BI-RADS4, and 248 lesions were included in this study and subsequently correlated with histopathological reports to calculate PPV. Since most breast cancer cases in SA were diagnosed at 45-55 years old, this study divided the patients into three groups: < 45 years, 45-55 years, and > 55 years. It correlates age subgroups and types of breast density to PPV. Exclusion criteria were non-Saudi, male patients, lesions without available images, or pathology results.

### Ethical Approval:

Ethical approval from the Institutional Review Board, General Directorate of Health Affairs in Madinah was obtained. National Registration Number with NICBE-KACST: (H-03-M-84, date August 23, 2022).

### Data handling, Imaging, and biopsy:

All suspicious lesions (classified as BIRADS4) were individually reviewed and scored as BIRADS 4A, 4B, and 4C blinded by an expert Breast Radiologist based on her experience in breast imaging (more than seven years) and ACR BI-RADS lexicon 5^th^ edition, regardless no particular objective criteria for BIRADS4 lesions, of which the evaluation was carried out based on mass’s shape (oval, lobular and irregular), suspicious calcification, architecture distortion and asymmetries on mammography as well sonographic features like orientation, margin, echo pattern, posterior features, vascularity, and skin thickening, etc.

In our institution, MG and US are performed routinely for diagnostic purposes in all women aged more than 35 years. US performed by breast radiologist on two machines, GE Healthcare, Wauwatosa, WI, USA and Philips, EPIQ 7G, USA, using a high-frequency linear probe (6-15 and 5-12 MHz respectively). MG was performed by an expert technologist who has been working for more than 16 years, using Hologic Selenia Dimensions, Hologic (USA), where Mediolateral oblique (MLO), craniocaudal (CC) views, and tomosynthesis obtained for all women’s complementary views as compression, magnification was performed when needed. Breast density type was assessed from MG images. All BI RADS4 lesions were sampled under US guidance using a 14 gauge core needle through an automated gun; at least three samples were taken from each lesion.

### Statistical Analysis:

IBM SPSS Statistics (version 23) was used for data entry and analysis. Categorical variables were expressed as frequencies and percentages. The Chi-square test was used to investigate any correlation between the variables. Statistical significance was defined as a p-value less than 0.05.

## RESULTS

Between September 2021 and June 2022, 3193 patients underwent MG ad US. There were 274 BI-RADS 4 lesions; of them, 16 were excluded due to lack of pathology results, and ten were excluded because there were no available images. The total number of patients was 248 The mean age was 44.62 ±14.32, ranging between 14 to 87 years.

Regarding the histopathological findings concerning BI-RADS four subcategories, 200 (81%) were reported as benign cases, 42 (17%) as malignant cases, and 6 (2%) as high-risk lesions. Regarding benign lesions, benign breast tissue (29%) was the most common histopathological type in subcategory 4A (33%). Fibroadenoma was the second most frequent histopathological finding in subcategory 4A (27%). Meanwhile, fibrocystic disease/change and fibroadenosis were the most common benign lesions categorized as BI-RADS 4B (24%). Also, regarding subcategory 4C, chronic mastitis was the most common histopathological type (seven patients, 54%). On the other hand, regarding the malignant lesions, invasive ductal carcinoma IDC 2 was the most common overall (40%) and also in each subcategory (40% in 4A, 75% in 4B, and 36% in 4C). This was followed by IDC 3 (36% overall, 40% in 4A, 25% in 4B, and 36% in 4C). Atypical ductal hyperplasia represented the high-risk lesions (100%), with two patients in each subcategory, Table-I.

**Table-I:** Histopathological findings in relation to BI-RADS 4 subcategories.

Histological Findings	BI-RADS Categories			

	4A	4B	4C	Overall

	n=152	n= 48	n= 48	n= 248
Benign (%)	n= 145(95.4)	n=42(87.5)	n= 13(27.1)	n=200(80.6)
Fibroadenoma	39(26.9)	3(7.1)	0	42(21)
Fibrocystic disease/changes/Fibroadenosis	24(16.5)	10(23.8)	2(15.4)	36(18)
Benign breast tissue	48(33.1)	9(21.4)	2(15.4)	59(29.5)
Usual ductal hyperplasia	6(4.1)	6(14.3)	1(7.7)	13(6.5)
Papilloma	3(2.1)	4(9.5)	0	7(3.5)
Chronic mastitis	3(2.1)	3(7.1)	7(53.8)	13(6.5)
Fibrosis/ fibrosis with calcification	7(4.8)	2(4.8)	0	9(4.5)
Scleroses / adenoids	11(7.6)	3(7.1)	0	14(7)
Benign other	4(2.8)	2(4.8)	1(7.7)	7(3.5)
Malignant (%)	n= 5(3.3)	n=4(8.3)	n=33 (72.9)	n=42(17.0)
IDC1	0	0	5(15.2)	5(11.9)
IDC2	2(40.0)	3(75.0)	12(36.4)	17(40.5)
IDC3	2(40.0)	1(25.0)	12(36.4)	15(35.7)
Malignant others	1(20.0)	0	4(12.0)	5(11.9)
High risk lesions (%)	n=2(1.3)	n=2(4.2)	n=2(4.2)	n=6(2.4)
Atypical ductal hyperplasia	2(100)	2(100)	2(100)	6(100)

Results are expressed as n (%); benign other include tubular adenoma, lactating adenoma, inclusion cyst, intraductal papilloma, myofibroplastoma, whereas malignant other includes ductal carcinoma in SITU, ILC, Mucinous carcinoma.

Out of the total of 248 cases. 132 patients (53%) had a left-sided tumour, while 116 (47%) had a right-sided tumour. Patients with benign lesions were significantly younger than those with malignant lesions (43.01±14.03 vs. 51.31±13.68, respectively; p-value<0.001). For both groups, most of the patients had scattered areas of fibroglandular density (32% of the benign and 40% of the malignant), followed by heterogeneously dense breast composition (28% of the benign and 37% of the malignant). In the benign group, the majority of patients were categorized as 4A (145 patients, 72%), while in the malignant group, the majority were in the 4C category (35 patients, 73%), Table-II.

**Table-II:** Characteristics of BI-RADS 4 category patients.

Characteristic	Benign (n=200)	Malignant (n=48)	P-value
Age (Mean ± SD)	43..01±14.03	51.31±13.68	P<0.001
Group-1 (< 45 years)	113(56.5)	15(31.3)	0.003
Group-2(45 -55 years)	48(24)	14(29.2)	
Group-3 (>55 years)	39(19.5)	19(39.6)	
***Breast Composition (%)***			
Entirely fatty	21(10.5)	7(14.6)	0.034
Scatter area of fibroglandualr density	65(32.5)	19(39.6)	
Heterogeneously Dense	56(28)	18(37.5)	
Extremely Dense	2(1)	1(2.1)	
No assessment (US only)	56(28)	3(6.2)	
***BI-RADS categories (%)***			
4A	145(72.5)	7(14.6)	<0.001
4B	42(21)	6(12.5)	
4C	13(6.5)	35(72.9)	

The results are presented in n (%) for categorical variables, Mean± SD (standard deviation) US; ultrasonography, BI-RADS; Breast Imaging Reporting and Data System, P-value <0.05 as statistical significance.

The age-related PPV of each BI-RADS category showed no significant differences among all age groups, both overall and within the subcategories. There were non-significant positive relationships between increasing age and age-related PPV: 3% vs.3% vs.10% for subcategory 4A (p-value=0.561); 5% vs. 8% vs. 29% for subcategory 4B (p-value=0.219); and 65% vs. 75% vs. 80% for subcategory 4C (p-value=0.969). More details regarding Age-related PPV cases in relation to BI-RADS 4 subcategories shows in Table-III. Moreover, there was no significant association between the age groups and BI-RADS 4 subcategories (p-value=0.516), Table-IV.

**Table-III:** Age-related PPV cases in relation to B_RADS 4 subcategories.

Age	BI-RADS Categories			

	4A	4B	4C	Overall

	n=7	n=6	n=35	n=48
Mean ± SD	49.14±16.04	56.83±12.67	50.80±13.53	51.31 ±13.68
***Age group (%)***				
Group-1 (< 45 years)	3.3(3/90)	4.8(1/21)	64.7(11/17)	10.9(14/128)
Group-2(45 -55 years)	3(1/33)	7.7(1/13)	75(12/16)	22.6(14/62)
Group-3 (>55 years)	10.3(3/29)	28.8(4/14)	80(12/15)	32.7(19/58)
Total (%)	4.6(7/152)	12.5(6/48)	72.9(35/48)	19.4(48/248)
ACR 2013 reference range (%)	>2 to <10	>10 to ≤50	>50 to <95	>2 to <95
P-value	0.561	0.219	0.969	0.645

Results are expressed as %(fraction); BI-RADS; Breast Imaging Reporting and Data System, SD, standard deviation, ACR, American College of Radiology. P-value <0.05 as statistical significance.

**Table-IV:** Association between the age group of PPV cases with B_RADS 4 subcategories.

Age group (%)	BI-RADS Categories			P-value

	4A	4B	4C	

	n=7	n=6	n=35	
Group-1 (< 45 years)	3	1	11	0.516
Group-2(45 -55 years)	1	1	12	
Group-3 (>55 years)	3	4	12	

Results are expressed as n; BI-RADS; Breast Imaging Reporting and Data System, P-value <0.05 as statistical significance.

The breast composition-related PPV cases with BI-RADS four subcategories differed significantly within subcategory 4C (p-value <0.001) and also overall (p-value<0.001) but showed no significant differences within subcategories 4A (p-value=0.934), and 4B (p-value=0.607). Table-V Regarding the ordinal logistic regression analysis, IDC2 was a significant reported 4B category of BI-RADS (SE=1.60; p-value <0.001). No other significant results were found, Table-VI.

**Table-V:** Breast composition-related PPV cases with B_RADS 4 subcategories.

Breast composition (%)	BI-RADS Categories			

	4A	4B	4C	Total
Entirely fatty	9.5(2/21)	60(3/5)	100(2/2)	25(7/28)
Scatter area of fibroglandualr density	2.4(1/41)	10(2/20)	69.5(16/23)	22.6(19/84)
Heterogeneously Dense	5.1(2/39)	6.6(1/15)	75(15/20)	24.3(18/74)
Extremely Dense	0(0/2)	0(0/0)	100(1/1)	33.3(1/3)
No assessment (US only)	4.1(2/49)	0(0/8)	50(1/2)	5.1(3/59)
Total	4.6(7/152)	12.5(6/48)	72.9(35/48)	19.4(48/248)
ACR 2013 reference range (%)	>2 to <10	>10 to ≤50	>50 to <95	>2 to <95
P-value	0.934	0.607	<0.001	<0.001

Results are expressed as %(fraction); BI-RADS; Breast Imaging Reporting and Data System, ACR, American College of Radiology. P-value <0.05 as statistical significance.

**Table-VI:** Results from ordinal logistic regression analysis to determine the effect of age on BI-RADS 4 subcategories.

Reference category	BI-RADS category	Variables in model	Statistic	SE	P-value
4A	4B	IDC1	18.770	1478	0.999
		IDC2	19.872	1.60	<0.001
		IDC3	18.110	0.000	NA
		Group-1 (< 45 years)	-1.850	1.67	0.269
		Group2 (45 -55 years)	-18.565	6068	0.998
	4C	IDC1	19.192	11326	0.999
		IDC2	.545	1.46	0.710
		IDC3	.633	1.41	0.653
		Group-1 (< 45 years)	.381	1.17	0.746
		Group2 (45 -55 years)	.991	1.37	0.470

Results are expressed statistic and standard error (SE); BI-RADS; Breast Imaging Reporting and Data System, P-value <0.05 as statistical significance.

## DISCUSSION

In this study, patients with benign lesions were significantly younger than those with malignant ones. There were gradually positive relationships between increasing age and age-related PPV in subcategories 4A-C. However, there was no significant difference. On the other hand, there was a significant difference within breast composition-related PPV cases with BI-RADS4 subcategories in subcategory 4C, and overall. In 4C, a total of 16 out of 23 had a scatter area of fibroglandualr density, and 15 out of 20 had heterogeneously dense breast composition. Few patients were included with entirely fatty (2/2) or extremely dense (1/1) breast composition. A significant relationship was observed between increased breast density and BIRADS 4C lesions. However, this could be related to improper sample distribution, and most malignant masses are BIRADS 4C. However, no other significant results were observed.

According to research published in 2019 by the National Breast and Ovarian Cancer Center of Australia, those with the highest mammographic breast density were shown to have a four to six-fold increased risk of breast cancer compared to women with average breast density. Recent reviews have indicated that this association is controversial.^10^ This unfavourable result may stem from the BI-RADS lexicon’s qualitative assessment fashion, emphasizing the possibility that dense breast tissue may mask a lesion rather than the amount of fibroglandular tissue that increases the risk of breast cancer.^10^ Another reason that caused difficult interpretation was fewer populations in the entirely fat (2%) and extremely dense (3%) types in comparison to other types of breast composition.^11^

On the other hand, a retrospective study conducted in southern Thailand of 924 patients found a significantly positive association between PPV and age in patients with lesions of BI-RADS subcategories 4 (A, B, and C).^11^ However, they could not conclude that breast composition through mammography, with regards to the ACR BI-RADS 5th edition, had associations with a PPV because of the improper distribution of the sample.^11^ Hu et al.^12^ also stated that the age-related PPV gradually increased with age for the 4A, B, and C subcategories. Meanwhile, a retrospective record-based study of 246 Indian patients concluded that BI-RADS 4A patients tended to be younger and had smaller mass sizes at presentation compared to BI-RADS 4 other subcategories (B and C).^13^ Also, digital mammography presented high predictability amongst BI-RADS 4 subcategories.^13^

The authors discussed that mammography reporting quality varies across the institutes and is not conclusive, as the predictive value of an abnormal mammogram might be higher in academic centres (three times) compared to community-based practices.^14^,^15^ Other factors that might explain the higher mammography’s predictability and sensitivity in their study might be their sole inclusion of diagnostic cases, resulting in overall higher mammography predictability.^13^ In addition, patients younger than 40 years within the BI-RADS 4A subcategory on mammograms might undergo extra non-invasive imaging with MRI, which might downscale the lesion category, subsequently decreasing unessential biopsy and surgery.^13^ Pape et al. did not find a correlation between BI-RADS category, age, and mammographic parenchymal patterns in Papua New Guinean women.^16^

In addition to this, Wang et al.^17^ found that the BI-RADS density classification, as well as Quantra measures, showed that, in Chinese women, mammographic density was positively associated with breast cancer risk and that these associations varied by age at menarche or first childbirth, parity, and duration of breastfeeding. The authors^17^ stated that the conventional BI-RADS density classification tool depends on the radiologists’ non-objective assessment, producing variable results and lacking inter- and intra-evaluator reliability.^18^,^19^ Also, it is affected by image characteristics as the evaluated images are made following the raw data reconstruction. Compared with the BI-RADS classification, the mammography density classification obtained from the raw data analysis using Quantra volumetric density measurement software had superior stability and repeatability.^20^

Hence, this software can accurately reflect the risk association between mammographic density and breast cancer.^17^ Meanwhile, a previous matched case-control study found that the BI-RADS density categorization by assigned radiologists had the same accuracy as computer-assisted methods (Cumulus and Volpara) for distinguishing breast cancer patients from the control.^21^ The age related-PPV for category 4, subcategories 4A and 4 C in all age groups, and 4B in age Group-3 (>55years) were within the ACR 2013 (the latest fifth edition) reference ranges, (4A=3 %, 3% 10% and in 4C= 65%, 75%, and 80% and in 4B in age Group-3 = 29%. It was lower than the ACR reference range, which is>2 to <10 in subcategory 4A, >10 to ≤50 in 4B, and >50 to <95 in 4C.^12^

In subcategories 4B in age Group-1 < 45 years = 5% and in Group-2 (45-55 years) = 8%, which is likely due to the low patients’ number in this group and subsequently affected the reliability. This is different from other studies by Noonpradej et al. and Fu et al., which showed significant differences in 4A and 4B subcategories, but the age-related PPV in age Group-3 exceeded the reference range in 4B= 51% and in 4A 16%, respectively.^11^,^22^ Other studies^23^-^25^ have confirmed the presence of significant differences in all subcategories 4A-C. However, PPV in age Group-3 (>50 years) was more than the reference range in 4B = 56% in Hu et al. and age Group-2 (45-55 years) in 4A in He et al.^24^ This discrepancy from other studies is possibly due to the different age groups used in our study which is related to the incidence of female breast cancer in Saudi Arabia. Additional possible explanations included isolated distinctive observations after sample size, the patient’s disposition, and selection bias. Furthermore, they discussed that these inconsistencies might be non-rigid BI-RADS recommendations leading to improper categorizations in BI-RADS 4 subgroups; for example, some of the lesions categorized as BI-RADS 4A might belong to BI-RADS 4B or 4C.^13^

This study’s most common malignant lesion was invasive ductal carcinoma, which is consistent with previous studies.^11^,^12^,^26^ On the other hand, the most typical benign lesion was benign breast tissue (29%), followed by fibroadenoma (21%), and fibrocystic disease/changes/fibroadenosis 18%), contrary to previous studies in which fibroadenoma was the most frequent benign lesion.^11^,^12^

### Strengths of the study:

It includes being the first in Saudi Arabia to study the effect of patient age and breast density on BIRADS 4 subcategorization, which shall open doors for future research.

### Limitations:

It include the retrospective nature of the study, small sample size, and improper sample distribution of breast density.

## CONCLUSION

To conclude, the study mainly found non-significant associations between the effect of patients’ age and breast parenchymal density on BI-RADS 4 subcategorization, except for significant differences within breast composition-related PPV cases with BI-RADS 4C sub subcategory. All BI RADS4 lesions should be given attention during practice regardless of age or breast composition.

## Data availability:

Upon request from the corresponding author.
